# Healthcare Warranty Policies Optimization for Chronic Diseases Based on Delay Time Concept

**DOI:** 10.3390/healthcare9081088

**Published:** 2021-08-23

**Authors:** Heng Zhao, Zixian Liu, Mei Li, Lijun Liang

**Affiliations:** 1 College of Management and Economics, Tianjin University, Tianjin 300072, China; zhaoheng100@tju.edu.cn (H.Z.); liuzixian@tju.edu.cn (Z.L.); limeitju@hotmail.com (M.L.); 2 School of Management, Tianjin University of Traditional Chinese Medicine, Tianjin 301617, China

**Keywords:** healthcare warranty, chronic diseases, delay time model, disease monitoring

## Abstract

Warranties for healthcare can be greatly beneficial for cost reductions and improvements in patient satisfaction. Under healthcare warranties, healthcare providers receive a lump sum payment for the entire care episode, which covers a bundle of healthcare services, including treatment decisions during initial hospitalization and subsequent readmissions, as well as disease-monitoring plans composed of periodic follow-ups. Higher treatment intensities and more radical monitoring strategies result in higher medical costs, but high treatment intensities reduce the baseline readmission rates. This study intends to provide a systematic optimization framework for healthcare warranty policies. In this paper, the proposed model allows healthcare providers to determine the optimal combination of treatment decisions and disease-monitoring policies to minimize the total expected healthcare warranty cost over the prespecified period. Given the nature of the disease progression, we introduced a delay time model to simulate the progression of chronic diseases. Based on this, we formulated an accumulated age model to measure the effect of follow-up on the patient’s readmission risk. By means of the proposed model, the optimal treatment intensity and the monitoring policy can be derived. A case study of pediatric type 1 diabetes mellitus is presented to illustrate the applicability of the proposed model. The findings could form the basis of developing effective healthcare warranty policies for patients with chronic diseases.

## 1. Introduction

Hospital readmissions have received substantial worldwide attention in recent years. According to a report, over 17% of patients were readmitted within 30 days of discharge [1]. Hospital readmissions are often costly, representing USD 25 billion per year [2]. However, approximately 75% of all readmissions can be prevented by appropriate post-discharge monitoring and management [3]. A high readmission rate imposes a heavy financial burden on patients but is also regarded as a symbol of poor quality of medical services [4,5,6].

To reduce readmissions and curb rising medical costs, there have been many payment systems, such as capitation, pay-for-performance, per diem prospective payment, and diagnostic-related groups, aiming at replacing the traditional fee-for-service (FFS) that has long been criticized for rewarding healthcare providers who spend more unnecessarily [7,8]. In 2013, the Centers for Medicare and Medicaid Services (CMS) initiated a new payment program called Bundled Payments for Care Improvement (BPCI) [9]. BPCI requires hospital to define bundles of healthcare services associated with a specified disease. For example, bundles for joint replacement include advance payments, details of healthcare services during the episode, and plans for post-discharge monitoring [10,11]. Under the bundled payment (BP) system, the hospital will receive a lump sum for a whole episode of care, including index admissions and readmissions that occur over a specified warranty period, such as 90 days after discharge, regardless of the treatment details and the possible related complications [12]. Here, BP is a reimbursement scheme with the implication of healthcare warranty, and the 90 days after discharge can be regarded as the warranty period. In addition to BP, there are other forms of payment such as ProvenCare and PROMETHEUS, which can be described as healthcare warranties [13]. Under the healthcare warranty, if the actual healthcare costs are lower than the lump sum, the hospital will profit; otherwise, the hospital will suffer a financial loss [14]. Therefore, the transformation of the payment mechanism from the traditional FFS to healthcare warranty has shifted the financial risk from the patient to the healthcare provider. Some scholars hold that healthcare warranty payments contribute to a higher quality of healthcare and keep treatment costs under control because patients would no longer pay for hospital readmissions when they occur [9,15]. Thus, providing a high level of treatment services to the patient during index admission may increase the profit margins by reducing the probability of readmissions under healthcare warranty payments [16]. The healthcare warranty reimbursement scheme is currently applied to joint replacement surgery, cardiovascular care, cancer care, and treatment of chronic diseases such as diabetes and chronic obstructive pulmonary disease [15,17,18,19,20,21]. The practice has proven that healthcare warranties can be greatly beneficial for reducing costs, improving patient satisfaction, and enhancing the competitiveness of medical institutions. Numerous efforts have been made to improve healthcare performance, from strategic to operational levels. Strategic decisions involve capital investment, resource planning of healthcare services, etc. [22,23]. Healthcare operations management focuses on the decision making related to the execution of healthcare delivery processes, including healthcare resources scheduling, process optimization and control, disease management, etc. [24,25,26]. Disease management comprises disease prediction and diagnosis, risk assessment, healthcare service strategy optimization, and disease monitoring [27,28,29,30]. In addition, research on healthcare cost control and management support systems in recent years has also created value in healthcare. For example, time-driven activity-based costing has been applied in healthcare and can help to efficiently modify cost processes and thereby contribute to cost control and efficiency improvement in healthcare delivery [31]. Another example is a healthcare management system with blockchain-based electronic healthcare records against the background of data security and inefficiently management [32]. This study is intended to provide an effective medical service decision-making model to enrich disease management research.

Jacobs et al. [33] showed that many types of patients’ post-discharge care processes and readmission characteristics are similar. For example, when a cystectomy patient is discharged from hospital, a disease-monitoring plan consisting of periodic follow-up is made. After the patient is discharged, they will receive scheduled follow-up checkups, such as a timely general inspection, comprehensive inspection, phone call, and office visit. The purpose of follow-up is to detect whether the patient has developed illnesses or complications prior to patient readmission. Once a condition is detected by follow-up, measures such as drug interventions can prevent further development. Moreover, early intervention and prevention programs may save total treatment costs and improve service quality simultaneously. Previous literature shows that undertaking a series of disease-monitoring plans can significantly reduce readmission rates [14,34]. Based on our field research, the patient’s condition goes through several states of progression before readmission. As time passes, the patient may develop a readmission-causing condition (called an “illness” in this article). When the illness first develops, it will not trigger immediate readmission. However, if nothing is done to address this illness, the patient’s condition will worsen and eventually lead to readmission. We call the time lapse between a detectable illness and readmission the delay time. In this study, we developed an optimization model based on a delay time model from the reliability and machine maintenance literature to capture disease-monitoring dynamics. The delay-time model is widely applied to model engineering problems in machine maintenance and inspection; it assumes that a device’s life cycle has an increasing failure rate [35,36,37]. The period that starts with the device showing signs of failure, and ending with the final failure, is called the delay time. In medical operations management, the delay-time model has been used to model disease progression. Fu et al. [38] proposed a two-stage delayed diagnosis regression model for hepatitis screening based on a delay time model. Zhang et al. [39] applied a delay time model to optimize postoperative monitoring plans for vascular surgery by minimizing the probability of failing to detect patency loss before readmission. Helm et al. [40] developed mixed integer programming based on a delay time model to determine optimal follow-up schedules and allocate necessary resources. Liu et al. [2] developed a delay time model to analyze the impact of quantity, effectiveness to detect conditions and a mix of different checkup methods on the optimal decision making of follow-up schedules.

Providing disease monitoring for patients implies additional costs for the hospital under the healthcare warranty, and these costs result from labor costs for medical staff, medical devices, and drugs during a predetermined warranty period. However, these interventions may lower the lump sum of healthcare warranties by reducing readmissions rates. The episodes of care can be divided into two consecutive periods: the hospitalization phase and the post-discharge phase. Healthcare providers need to develop a healthcare warranty policy that includes treatment decisions for the first phase and monitoring plans for the second phase. Treatment decisions affect the baseline readmission rate after discharge, and the follow-up monitoring strategy will also affect the readmission rate. Although a high number of treatment decisions incur higher medical costs, providing a high number of treatment options may reduce overall costs by reducing baseline readmission rates. Therefore, for cost control and profitability, joint optimization of hospital treatment decisions and monitoring strategies is of great significance for reducing patient readmissions and controlling the healthcare costs for a medical institution.

Previous studies and medical practices have demonstrated the potential of a healthcare warranty in terms of cost saving and quality improvement [9,14,15]. However, some questions need to be further explored, which are also the motivation for this study. First, existing studies on disease-monitoring policies have aimed at maximizing the probability of detecting the patients’ health conditions but failed to consider the costs associated with follow-up and treatment. Sujan et al. [41] pointed out that it is necessary for hospitals to establish a mechanism for managing explicitly the trade-off between risk reduction and cost. Second, the extant research does not use appropriate models to simulate the effect of disease monitoring on the patients’ readmission rates. The purpose of disease monitoring is to reduce the readmission rates by follow-up checkups of patients to detect the illness early and take intervention measures to remove it. Consequently, after an illness intervention, the progress of the disease is expected to be halted, and the readmission risk of the patient is expected to be reduced compared to that before the follow-up interventions, which may in turn influence the decisions made about a disease-monitoring policy. In addition, although healthcare warranties are beneficial for cost savings and medical quality assurance, the method behind designing effective healthcare warranty policies is poorly understood. Currently implemented healthcare warranty policies have been obtained empirically and subjectively without supporting quantitative analysis. 

Our study is relevant to the research on developing the optimal follow-up policies based on the delay time model as mentioned above [2,39,40]. Given that a patient develops a readmission-causing condition after discharge, the delay time defines a time window during which a follow-up checkup can prevent readmission. Existing research uses the delay time model to characterize the dynamics of readmission and further derives the optimal follow-up schedule to maximize the probability of detecting the readmission-causing condition. Our research extended these modeling approaches in several aspects. First, we considered the impact of inpatient treatment decisions on baseline readmission risk and optimized treatment decisions and follow-up monitoring strategies simultaneously. Second, we measured the effect of follow-up intervention on the patient’s readmission risk. Both extensions made the model construction and computational complexity more challenging. The main contributions are as follows. We proposed a systematic optimization model of a healthcare warranty policy that allows healthcare providers to determine the optimal combination of treatment decisions and disease-monitoring policies to minimize the total expected healthcare warranty cost over the prespecified period. To the best of our knowledge, there has been a lack of research on the development of healthcare warranty policies in the healthcare operations and management. We used elaborate mathematical formulas to characterize the relationship between the treatment intensity and baseline readmission rates, and we utilized the delay time model to analyze and optimize the disease-monitoring strategy. The effect of a follow-up intervention on the patient’s readmission risk was measured by the accumulative age method. In addition, a healthcare warranty policy optimization model was constructed to determine the optimal combination of the treatment decisions and the timing of follow-up checkups during the warranty period. A case study of pediatric type 1 diabetes mellitus is presented to illustrate the applicability of the proposed model. The proposed model framework and practical implications can provide a basis for the healthcare provider to formulate healthcare warranty policies, further contributing to improving the quality of healthcare and control the costs. Furthermore, the application of the delay time model and accumulative age method provide a reference for disease modeling and management.

## 2. Problem Definition

**Figure 1:**
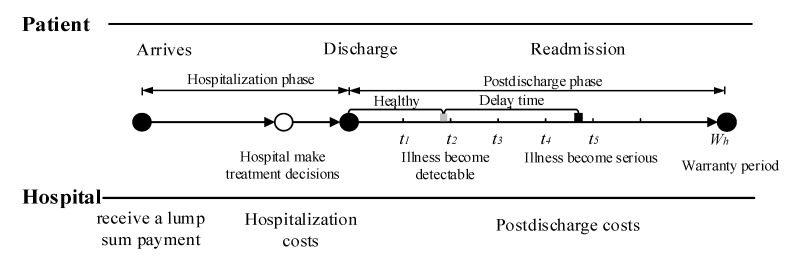
Sequence of events.

Consider a healthcare delivery system with two parties, namely, a hospital and its cohort of patients. Under the healthcare warranty scheme, the length of care episodes (i.e., warranty period) is prespecified. Once a patient enters the hospital, the hospital receives a lump sum payment to cover all services during the entire care episode. These medical services include treatment options during hospitalization, disease monitoring, and possible readmission treatment after discharge. Figure 1 illustrates the sequence of events. In medical practice, patients are often considered to be homogeneous under healthcare warranties. This consensus is reasonable, since patients are classified into heterogeneous diagnosis-related groups (DRGs) based on their individual characteristics. Patients in the same group have similar physical and disease conditions; that is, they are homogeneous [42,43]. When a patient is entered into a healthcare delivery system, the hospital chooses treatment intensity $\theta \in \left[\theta_{min},\theta_{max}\right]$ to maximize their expected profit [14]. The variable θ refers to the level of treatment services provided by the hospital for patients, such as the professional level of doctors, the professional competence of the nursing staff, the length of stay (LOS), and the precision of the medical devices. The associated costs incurred by hospitals during hospitalization increase with the treatment intensity θ. After a patient is discharged, disease-monitoring plans consisting of periodic follow-up activities are necessary to reduce hospital readmission rates and control healthcare costs. During the post-discharge phase, the patient’s condition undergoes several states of progression before readmission. In this study, we used a delay time model to capture the progression of disease. For example, diabetic patients usually have polydipsia and polyuria before being readmitted to the hospital. Under the condition that the patient is monitored after discharge from the hospital, if any readmission-causing conditions are detected by follow-up, intervention measures can be taken immediately to mitigate illness deterioration. The purpose of disease monitoring is to detect developing conditions before they deteriorate, reducing unnecessary readmissions.

## 3. Mathematical Model

In this section, we first presented the model notations and assumptions to make the mathematical model tractable. After that, we constructed a two-phase optimization model including the hospitalization phase and post-discharge phase under healthcare warranties.

### 3.1. Notations and Assumptions

The notation is introduced as follows.
θTreatment intensity for a patient during hospitalization, $\theta \in \left[\theta_{min},\theta_{max}\right]$
$C_{T}\left(\theta \right)$Treatment cost incurred by hospital during a patient’s hospitalizationTFollow-up interval$t_{i}$Follow-up instants, (i=1,2,…)
$c_{f}$Average cost of each follow-up$c_{d}$Average cost of each drug intervention triggered by follow-up$c_{r}$Average cost of each readmission$W_{h}$Prespecified length of warranty perioduTime lapse between latest follow-up and the illness being detectablevTime lapse between the illness being detectable and readmission$f_{0}\left(u,\theta \right)$Baseline probability density function (pdf) of illness$\lambda_{0}\left(u,\theta \right)$Baseline instantaneous rates of illnessh(v)Instantaneous rates of readmission$\tau_{k}$The time of the kth drug intervention ρImprovement factor of drug intervention (0≤ρ≤1)ΔiAccumulative age after drug intervention at $t_{i}$
$\lambda_{i+1}\left(u,\theta \right)$Instantaneous rate of illness after the drug intervention at time $t_{i}$
$f_{i+1}\left(u,\theta \right)$Probability density function (pdf) of illness after the drug intervention at $t_{i}$
$F_{i+1}\left(u,\theta \right)$Cumulative distribution function (cdf) of illness after the drug intervention at $t_{i}$
g(v)Probability density function (pdf) of readmissionG(v)Cumulative distribution function (cdf) of readmission

The following assumptions are taken into account:

- Patients are discharged healthy.
- The time to illness becoming detectable post-discharge is a random variable following a Weibull distribution [2,44].
- Follow-ups are periodic and can detect the patients’ condition perfectly. Once a patient is detected to be in an illness state, a drug intervention is carried out to immediately resolve any illness.
- The effectiveness of drug intervention triggered by follow-up is imperfect, while readmission treatment is perfect. This assumption is reasonable in medical practice because the patient’s physical condition at the time of discharge needs to meet discharge criteria [45].
- The duration of follow-up and readmission treatment is negligible compared with the prespecified length of the warranty period [46].

### 3.2. Hospitalization Phase

The treatment intensity θ is used to quantify treatment decisions during the hospitalization period. When a patient is accepted by a hospital, the hospital determines treatment intensity $\theta \in \left[\theta_{min},\theta_{max}\right]$ for the patient to maximize its expected profits. For example, for the treatment of patients with chronic gastropathy disease, there are five different treatment intensities: (1) routine visit and consultation, (2) daily dose prescription omeprazole 20 mg tablet for a period of 30 days, (3) manometry, (4) endoscopy, and (5) Laparoscopic Nissen Fundoplication (LNF) surgery, where each treatment level is cumulative (for example, level 2 is to prescribe the drug and do a routine consultation). To calculate the treatment cost during the hospitalization period, Adida et al. [14] used a simple power function of treatment level to characterize the treatment costs under different treatment levels. However, their model cannot clearly reflect the cost component because treatment costs depend almost entirely on the treatment intensity. In this study, we assumed that with the increase in treatment decision variable θ, the speed of marginal treatment cost increases. Therefore, $C_{T}\left(\theta \right)$ is increasing and convex in θ. This is more accurate than a simple power function to calculate the costs, but it inevitably increases the computational complexity. The treatment cost during the hospitalization period is expressed as follows:

(1) $$C_{T}\left(\theta \right)=A_{0}+A_{1}\;exp\left(k\frac{\theta_{max}-\theta }{\theta -\theta_{min}}\right)$$

where θ is the treatment intensity parameter ($\theta_{min}<\theta \leq \theta_{max}$), $A_{0}$ represents the basic costs, and $A_{1}$ is the variable costs associated with the treatment intensity.

### 3.3. Post-Discharge Phase

After hospitalization, patients are discharged from hospital at $t_{0}$. After discharge, the patient’s condition may worsen, eventually leading to readmission. However, when the illness first develops (e.g., an infection), it is not so serious that it can only be detected by a follow-up checkup. Additionally, it can hardly result in immediate readmission because there is a delay between the illness becoming detectable and readmission. To reduce the possibility of readmission, disease-monitoring plans must be developed for detecting illnesses early. In this study, the patient’s condition after discharge was monitored by periodic follow-up. The purpose of follow-up is to detect the illness before gradual deterioration and readmission. Once a condition is detected early by a follow-up, intervention measures (e.g., drug interventions) are carried out to resolve the illnesses. Our goal is to develop an optimal follow-up schedule under healthcare warranties.

#### 3.3.1. Discrete Follow-Up and Drug Intervention Actions

In this study, follow-up was assumed to be perfect for detecting patients’ conditions, while the effectiveness of drug intervention was imperfect. To model the effect of drug intervention, borrowing from maintenance techniques was used to describe the effectiveness of maintenance activities, we assumed that the *k*th drug intervention will reduce the length of the last drug intervention time from $\tau_{k}-\tau_{k-1}$ to $\rho \left(\tau_{k}-\tau_{k-1}\right)$, where $\tau_{k}$ is the time of the *k*th drug intervention and ρ=1−δ(m) is an improvement factor. Each drug intervention action reduces the “effective age” of the patient. The reduction in the effective age depends on the degree of intervention *m*, with δ(m) decreasing with *m* [47].

(2) $$\delta \left(m\right)=\left(1+m\right)e^{-m},0\leq \delta \left(m\right)\leq 1,\;0\leq m\leq M.$$

where δ(0)=1 (*m* = 0, no intervention measures are taken) and δ(M)=0 ( m=M, patient restored to the state of the last drug intervention).

For an infinite drug intervention effort *M* (impossible to implement in the actual treatment), the condition of the patient was restored to the same condition as it was following the previous drug intervention (δ(∞)=1). Hence, in this study, our model allows the drug intervention to be imperfect. 

**Figure 2:**
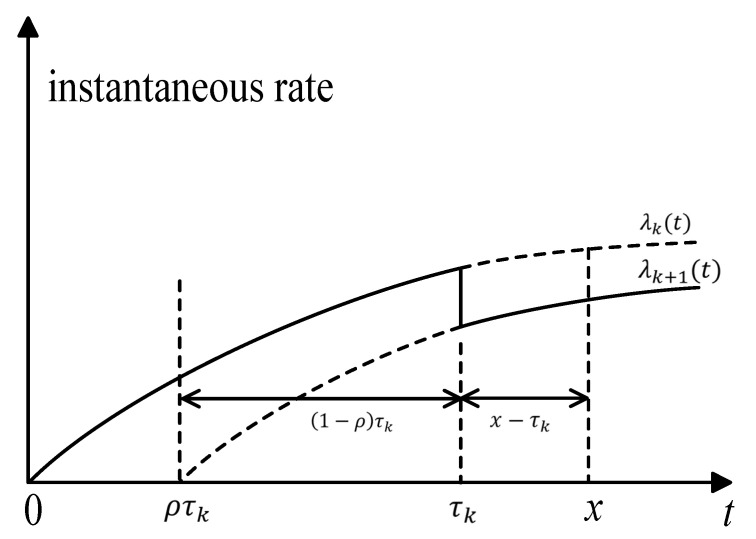
Illustration of the accumulative age.

For $x>\tau_{k}$, the effective age after the *k*th drug intervention is $v\left(x\right)=x-\rho \tau_{k}$. The instantaneous rate of illness after the *k*th drug intervention is $\lambda_{0}\left(v\left(x\right),\theta \right)$, where $\lambda_{0}\left(t,\theta \right)$ is the instantaneous rate of the illness without any drug intervention. In this paper, we use the concept of “accumulated age” to denote the accumulative deterioration of previous intervention actions; Figure 2 illustrates the accumulative age. The lapse of time between the *x* and the *k*th drug intervention is $x-\rho \tau_{k}$; in other words, the interval of $\left(1-\rho \right)\tau_{k}=x-\rho \tau_{k}-\left(x-\tau_{k}\right)$, is the accumulated age, which denotes the accumulated deterioration of previous intervention actions. The magnitude of accumulated age affects the instantaneous rate of illness in the (*k* + 1)th intervention stage.

Let $t_{i}$ be the time of the ith follow-up. In medical practice, follow-up activities are always carried out according to physicians’ experience or based on a fixed interval of *T*. For example, in the 2018 Diabetes clinical guidelines, the International Society for Pediatric and Adolescent Diabetes (ISPAD) recommends three-month follow-ups for HbA1c measurement for adolescents with diabetes. In this study, the time of the *i*th follow-up is $t_{i}=iT$. It is worth noting that not all follow-ups will induce drug intervention; hence, the time of *i*th drug intervention $\tau_{i}$ may be greater than $t_{i}$. If a drug intervention is triggered by the ith follow-up at $t_{i}$, then the accumulative age is:

(3) $$∆i=\left(1-\rho \right)t_{i}.$$

The instantaneous rate and the pdf of illness after the intervention action at $t_{i}$ is:

(4) $$\lambda_{i+1}\left(u,\theta \right)=\lambda_{0}\left(u+\Delta i,\theta \right)\;\mathrm{where}\;u=x-t_{i}.$$

The pdf and the cdf of illness after the intervention action at $t_{i}$ are:

(5) $$f_{i+1}\left(u,\theta \right)=\lambda_{i+1}\left(u,\theta \right)e^{-\int_{0}^{u}\lambda_{i+1}\left(s,\theta \right)ds}=\lambda_{0}\left(u+\Delta i,\theta \right)e^{-\int_{\Delta i}^{u+\Delta i}\lambda_{0}\left(s,\theta \right)ds}$$

(6) $$F_{i+1}\left(u,\theta \right)=1-e^{-\int_{\Delta i}^{u+\Delta i}\lambda_{0}\left(s,\theta \right)ds}.$$

Moreover, the pdf and the cdf of readmission are defined as:

(7) $$g\left(v\right)=h\left(v\right)e^{-\int_{0}^{v}h\left(t\right)dt}$$

(8) $$G\left(v\right)=1-e^{-\int_{0}^{v}h\left(t\right)dt}.$$

#### 3.3.2. Probability of Drug Intervention 

**Figure 3:**
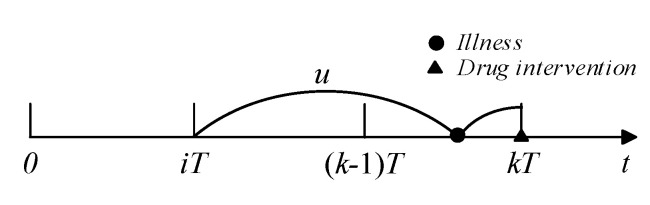
Illustration of drug intervention.

For the *k*th follow-up, if illness was detected and did not cause readmission, drug intervention actions could be taken to curb disease progression. If the last drug intervention was conducted at $t_{i}(i<k-1)$, the illness was developed at [(k−1)T,kT] and did not cause readmission (see Figure 3), and the probability of drug intervention occurring at kT can be calculated as:

(9) $$P\left[\left(k-i-1\right)T<u<\left(k-i-1\right)T\cap u+v>\left(k-i\right)T\right]=\int_{\left(k-i-1\right)T}^{\left(k-1\right)T}f_{i+1}\left(u\right)\left[1-G(\left(k-i\right)T-u\right)]du.$$

The probability of the last drug intervention occurring at $t_{i}$ is $P_{d}\left(i,T\right)$; thus, average probability that a drug intervention action occurs at $t_{k}$ is:

(10) $$P_{d}\left(k,T\right)=\sum_{i=0}^{k-1}P_{d}\left(i,T\right)\int_{\left(k-i-1\right)T}^{\left(k-1\right)T}f_{i+1}\left(u\right)\left[1-G(\left(k-i\right)T-u\right)]du,\;\mathrm{where}\;P_{d}\left(0,T\right)=1,\;k=1,\;2,\;\ldots$$

#### 3.3.3. Probability of Readmission

**Figure 4:**
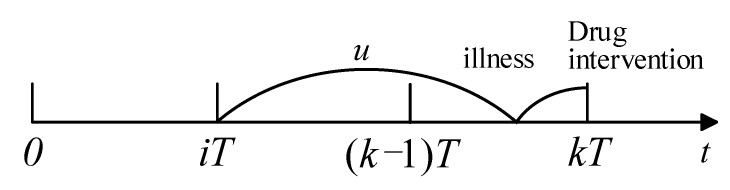
Process of readmission.

Similarly, if readmission happens in [(k−1)T,kT] and the last drug intervention was taken at iT, the illness was developed in [(k−1)T,kT] and caused readmission before kT (see Figure 4). Recalling a recent drug intervention at iT, the probability of readmission in [(k−1)T,kT] is

(11) $$P_{r}\left(k,T\right)=\sum_{i=0}^{k-1}P_{d}\left(i,T\right)\int_{\left(k-i-1\right)T}^{\left(k-1\right)T}f_{i+1}\left(u,\theta \right)G\left[\left(k-i\right)T-u\right]du.$$

If a patient has not been readmitted until *x* (mT<x<(m+1)T), this means that the patient did not develop an illness at *x*, or they have developed an illness at [mT, x] but the illness did not cause readmission before *x*. Thus, the average probability of survival is

(12) $$R\left(x,T\right)=\sum_{i=1}^{m}P_{d}\left(i,T\right)\left\{1-\int_{0}^{x-iT}f_{i+1}\left(u\right)du+\int_{\left(m-i\right)T}^{x-iT}f_{i+1}\left(u\right)\left[1-G(x-iT-u\right)]du\right\}.$$

#### 3.3.4. Cost Model

It is assumed that the cost of follow-up, drug intervention, and readmission treatment are independent of the severity of the patient’s condition. Readmission treatment is assumed to be so perfect so as to restore the patient’s condition to the state when they were first discharged; hence, the time lapse between two adjacent readmissions can be regarded as a renewal cycle. The time of follow-up, drug intervention, and readmission treatment can be neglected in our assumption. The expected length of a renewal cycle is calculated based on the following formulation:

(13) $$EL\left(T\right)=\int_{0}^{\infty }R\left(x,T\right)dx.$$

If a renewal cycle is terminated at [(k−1)T,kT], then the total cost during a renewal cycle is

(14) $$C\left(k,T\right)=\left(k-1\right)c_{f}+\sum_{i=1}^{k-1}P_{d}\left(k,T\right)c_{d}+c_{r}.$$

Since the occurrence of readmission may occur in any follow-up interval, the expected total cost per cycle over an infinite period is

(15) $$EC\left(T\right)=\sum_{k=1}^{\infty }C\left(k,T\right)P_{r}\left(k,T\right).$$

and therefore, the average expected cost during the post-discharge period is

(16) $$EC\left(W_{P},T\right)=\frac{EC\left(T\right)}{EL\left(T\right)}W_{h}=\frac{\sum_{k=1}^{\infty }C\left(k,T\right)P_{r}\left(k,T\right)}{\int_{0}^{\infty }R\left(x,T\right)dx}W_{h}.$$

### 3.4. The Optimization Model of Healthcare Warranty Policies

Under healthcare warranties, the expected total cost from a hospital’s perspective over the planning horizon depends on the treatment intensity *θ* and the follow-up period *T*. Our goal is to optimize both the treatment intensity and the follow-up period to control healthcare expenditures. The objective function of the healthcare warranty policy optimization model is given as:

(17) $$ECT=C_{T}\left(\theta \right)+EC\left(W_{P},T\right).$$

## 4. Case Study

### 4.1. Numerical Example

In this section, we perform numerical analyses on pediatric type 1 diabetes mellitus (T1DM). The primary goal of this research is to verify the feasibility of the proposed model and provide healthcare providers with a cost model and optimization method for healthcare warranty policies. T1DM is a condition resulting from the pancreas’ inability to produce insulin, and it is characterized by elevated blood glucose. Diabetes requires long-term comprehensive treatment and care, including insulin injections, diet control, and exercise. In addition, complications related to diabetes, such as diabetic ketoacidosis and hypoglycemia, increase readmission rates [48]. The dynamics of T1DM readmission occur as follows. First, the hospital chooses a treatment decision at $\theta \epsilon \left[\theta_{min},\theta_{max}\right]$ for patients and incurred cost of treatment $C_{T}\left(\theta \right)$ for the first stage. After the patient is discharged, the doctor will advise the patient to have an office visit every three months to see if they have developed a readmission-causing illness. The International Society for Pediatric and Adolescent Diabetes (ISPAD) also recommends that the pediatric and adolescent diabetics have a follow-up every three months. When the illness first develops, it is usually characterized by polydipsia, polyphagia, polyuria, and weight loss; however, it is not so serious enough to cause immediate readmission. During an office visit, routine laboratory tests for the HbA1c level and thyroid and endocrine functioning will be performed, as well as an inquiry for physical illnesses. If an illness has developed and is detected in the office visit, intervention measures, such as insulin injection and healthy guidance for patients, can be taken to control the disease progression. However, in cases where no examination and intervention measures are taken, the illness will worsen over time and eventually become so severe that the patient will be readmitted to the hospital. We called the period between illness detection and readmission the delay time.

Based on our field research, we considered that the interval between discharge and the illness being detected follows a Weibull distribution with a shape parameter *β_1_* and scale parameter *θ* where the scale parameter *θ* is regarded as the treatment intensity during the hospitalization phase. The delay time follows a Weibull distribution with shape parameter *β_2_* and scale parameter α. Let *β_1_* = 1.6, *β_2_* = 1.1, α = 0.08,$\;\theta_{min}=0.1,\;\;\theta_{max}=0.2$. The cost-related parameters used for the numerical experiment were based on an average of the costs associated with treatment pediatric and adolescent diabetes patients in a large general hospital in Tianjin, as shown in Table 1.

We conducted numerical experiments to calculate the optimal combination of treatment decisions and monitoring strategies under healthcare warranties. The healthcare warranty period was assumed to be two years. When patients are discharged from the hospital, monitoring plans consisting of periodic follow-up checkups are implemented to reduce patient readmissions. The optimal disease-monitoring plan and treatment decision are shown in Table 2. For the sake of calculation, the minimum unit of treatment intensity was fixed at 0.01 in the model. It can be observed from Table 2 that the optimal follow-up schedule was to conduct an office visit every three months; the total expected healthcare warranty costs (including treatment costs and follow-up costs and expected costs of readmission within the warranty period) were 11,294. 

### 4.2. Sensitivity Analysis

Sensitivity analysis is performed to investigate the effect of model parameters on the optimal solutions and the interaction between the treatment decision and disease-monitoring strategy. The parameters that we are interested in include (a) the average cost of each readmission $c_{r}$, (b) the follow-up cost $c_{f}$, and (c) the effectiveness of follow-up ρ. 

**Figure 5:**
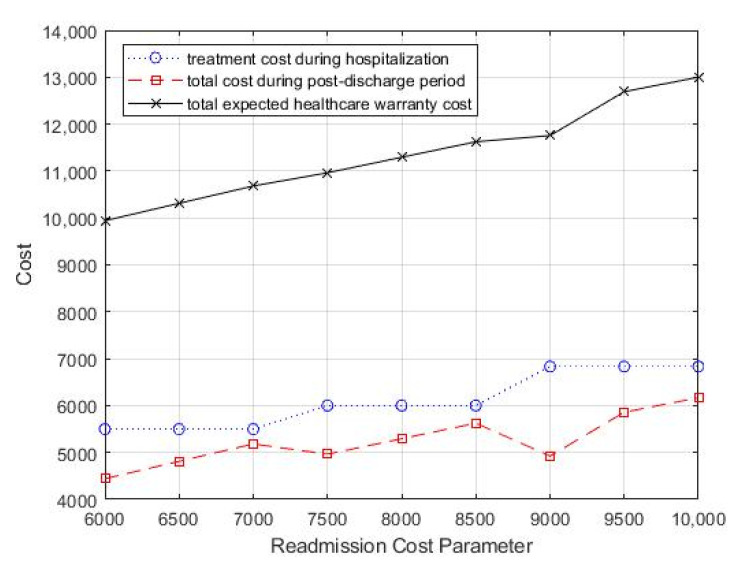
Change in readmission cost parameter, *c_r_*, for total costs.

The effects of varying the average cost of readmission $c_{r}$ on the optimal treatment decision and monitoring strategy, as well as the total expected costs, are presented in Table 3 and Figure 5. Table 3 shows that as the average cost of readmission increased, the optimal treatment intensity increased gradually, which means that more aggressive treatment during hospitalization is needed to improve the patient’s discharge prognosis to reduce potential readmission. In addition, as the readmission treatment cost increased, the value of T decreased gradually, which suggested that more frequent follow-up is necessary to reduce the total healthcare warranty cost. 

**Figure 6:**
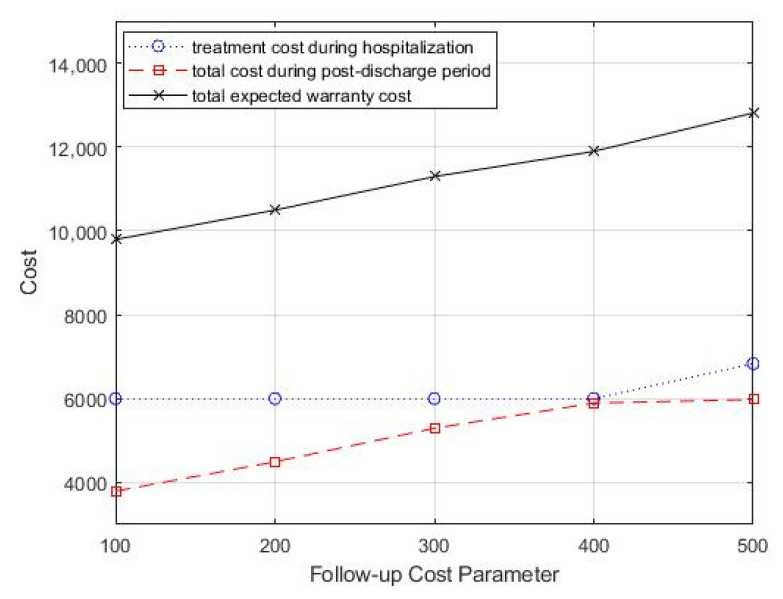
Change in follow-up cost parameter, *c_f_*, for total costs.

In the case study, the monitoring strategy consisted of periodic follow-up office visits, and each office visit incurred associated costs, including inspection costs, testing costs, and labor costs. Then, we analyzed the impact of follow-up costs on the optimal treatment decision and monitoring strategy by varying the follow-up costs *c_f_*. We started with a follow-up cost of 100 and increased it by 100. Table 4 summarizes the optimal combination of treatment decisions and monitoring strategy. From Table 4, we can see that as the follow-up cost increased, the optimal treatment intensity increased, but the optimal follow-up period did not show a significant variation pattern. In addition, the total healthcare warranty cost also showed an increasing trend. Figure 6 shows the change in total costs as the follow-up cost parameter changed.

**Figure 7:**
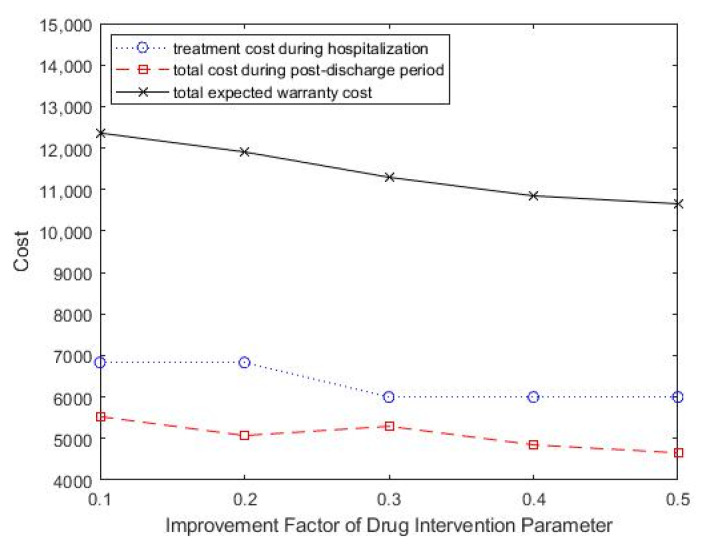
Change in improvement factor parameter of drug intervention, *ρ*, for total costs.

The effects of the improvement factor of drug intervention in reducing readmission performance changes on optimal treatment decisions and treatment intensity was also studied. As shown in Table 5, as the improvement factor of drug intervention increased, the optimal treatment intensity showed a downward trend. The total expected healthcare warranty cost also decreased, which suggested improved medical technology and effective drug development. Figure 7 shows the change in total costs as the improvement factor parameter of drug intervention change.

## 5. Discussion

### 5.1. Principal Findings

Healthcare warranties can be greatly beneficial to reduce costs, improve care quality, and enhance the competitiveness of medical institutions. Although existing studies have proved the advantages of a healthcare warranty in theory and practice, currently implemented healthcare warranty policies are obtained empirically and subjectively without supporting quantitative analysis [9,14,15]. The optimization model of healthcare warranty policies presented in this paper contributes new insights to healthcare operation and management.

The proposed model achieved the trade-off between treatment decisions and postdischarge monitoring strategies from a systematic point of view. Compared to the traditional subjective experience-based approach, the proposed framework realized optimal allocation of resources and avoided overspending and loss of healthcare providers. 

Since there is a time lapse between a readmission-causing condition and a readmission, we used a delay time model to simulate the progression of chronic diseases. To capture the effectiveness of the drug intervention on disease progression, the accumulated age model was employed. Currently, few studies have formulated the post-discharge monitoring strategy from the perspective of healthcare cost based on the delay time model [2,39], and there is little research on healthcare warranty policies that is based on analytical methods. This study fills this gap by constructing a healthcare warranty policy optimization model and providing new technologies and methods for healthcare research. The proposed model forms the basis for developing effective healthcare warranty policies for patients with chronic diseases.

### 5.2. Practical Implications

The healthcare sector has long been plagued by rising healthcare costs, which have put great pressure on the society and the government. Providing warranties for certain diseases contributes to care quality improvement and cost control. This study systematically optimized treatment decisions and follow-up strategies for healthcare warranties, and it has great potential for assisting healthcare providers to reduce healthcare risks and the associated costs. The healthcare warranty costs are usually paid by the insurer, and the proportion of the payment depends on the type of medical insurance that the patient purchased. Therefore, the actual cost savings can reduce the expenses of the insurer and promote the implementation of the healthcare warranty management.

The computational result of the proposed model in pediatric type 1 diabetes mellitus cases resulted in the optimal healthcare warranty policy, that is, the combination of treatment decision and monitoring strategy. The subsequent sensitivity analysis provided further guidance for healthcare providers to develop flexible healthcare warranty policies because it revealed the impact of key parameters on healthcare warranty performance. Application of the proposed model and its findings has the potential for reducing readmissions, improving healthcare quality, and reducing healthcare costs.

### 5.3. Limitations and Future Research

Despite the above theoretical and practical implications, this study has some limitations. First, the duration of readmission treatment and follow-up in this study was assumed to be instantaneous. Further research can modify this assumption by using random variables representing the checkup and treatment times. Second, the disease-monitoring strategy is consisted of periodic follow-ups. However, adjustments to the disease-monitoring strategy are needed, since the patient’s disease progression is dynamic and complex. In this case, dynamic follow-up policies are more applicable and need to be investigated, which may require the introduction of wearable devices [44]. Third, the proposed model is not suitable for all diseases. Our model focused on the certain diseases, especially chronic diseases with delayed readmission dynamics. In future research, it will be fruitful to extend the model to the design of healthcare warranty policy for other diseases according to their characteristics.

## 6. Conclusions

This study presented a healthcare warranty strategy optimization model for determining simultaneously the optimal combination of treatment decisions and the disease-monitoring strategy for patients with chronic diseases, which is aimed at minimizing the healthcare provider’s total expected warranty cost over a prespecified warranty period. We modeled the dynamics of chronic disease progression using the delay time model. The accumulated age model was employed to capture the effectiveness of the drug intervention on disease progression. To verify the feasibility and effectiveness of the proposed model, a case study of diabetes patients was presented. According to the proposed model, the optimal combination of treatment decisions and disease-monitoring strategies was calculated. The model proposed in this paper helps to enrich the healthcare warranty theory, including the formulation of healthcare warranties and the analysis of warranty costs. In addition, the proposed model can also be extended to warranty management for other chronic diseases. The application of the proposed model has the potential to reduce healthcare costs and improve care quality.

## Figures and Tables

**Table 1:** Parameters for the numerical example.

*A*_0_	*A*_1_	*k*	*c_f_*	*c_d_*	*c_r_*	*ρ*
4000	1000	1	300	150	8000	0.3

**Table 2:** The optimal disease-monitoring strategy and treatment decision for warranty period $W_{h}$.

$W_{h}$	θ	T	$C_{T}\;\left(\theta \right)$	$EC\;\left(W_{P},T\right)$	ECT
2	0.14	3	6000	5294	11,294

**Table 3:** Effect of readmission cost on the optimal treatment decision and monitoring strategy.

*c_r_*	θ	T	$C_{T}\;\left(\theta \right)$	$EC\;\left(W_{P},T\right)$	ECT
6000	0.15	3.5	5500	4441	9941
6500	0.15	3.5	5500	4812	10,312
7000	0.15	3	5500	5182	10,682
7500	0.14	3	6000	4963	10,963
8000	0.14	3	6000	5294	11,294
8500	0.14	3	6000	5625	11,625
9000	0.13	3	6833	4924	11,757
9500	0.13	2.5	6833	5859	12,692
10000	0.13	2.5	6833	6168	13,001

**Table 4:** Effect of follow-up cost on the optimal treatment decision and monitoring strategy.

*c_f_*	θ	T	$C_{T}\;\left(\theta \right)$	$EC\;\left(W_{P},T\right)$	ECT
100	0.14	2.5	6000	3794	9794
200	0.14	3	6000	4494	10,494
300	0.14	3	6000	5294	11,294
400	0.14	3.5	6000	5894	11,894
500	0.13	3	6833	5977	12,810

**Table 5:** Effect of improvement factor of drug intervention on the optimal treatment decision and monitoring strategy.

*ρ*	θ	T	$C_{T}\;\left(\theta \right)$	$EC\;\left(W_{P},T\right)$	ECT
0.1	0.13	2.5	6833	5528	12,361
0.2	0.13	3	6833	5072	11,905
0.3	0.14	3	6000	5294	11,294
0.4	0.14	3	6000	4847	10,847
0.5	0.14	3.5	6000	4655	10,655

## Data Availability

The data presented in this study are available on request from the corresponding author.
